# Effects of Continuous Erythropoietin Receptor Activator in Sepsis-Induced Acute Kidney Injury and Multi-Organ Dysfunction

**DOI:** 10.1371/journal.pone.0029893

**Published:** 2012-01-03

**Authors:** Camila E. Rodrigues, Talita R. Sanches, Rildo A. Volpini, Maria H. M. Shimizu, Patrícia S. Kuriki, Niels O. S. Camara, Antonio C. Seguro, Lúcia Andrade

**Affiliations:** 1 Department of Nephrology, University of São Paulo School of Medicine, São Paulo, São Paulo, Brazil; 2 Department of Nephrology, Paulista School of Medicine, Federal University of São Paulo, São Paulo, Brazil; 3 Department of Immunology, University of São Paulo, São Paulo, Brazil; National Cancer Institute Staff Scientist Mouse Cancer Genetics Program, United States of America

## Abstract

**Background:**

Despite advances in supportive care, sepsis-related mortality remains high, especially in patients with acute kidney injury (AKI). Erythropoietin can protect organs against ischemia and sepsis. This effect has been linked to activation of intracellular survival pathways, although the mechanism remains unclear. Continuous erythropoietin receptor activator (CERA) is an erythropoietin with a unique pharmacologic profile and long half-life. We hypothesized that pretreatment with CERA would be renoprotective in the cecal ligation and puncture (CLP) model of sepsis-induced AKI.

**Methods:**

Rats were randomized into three groups: control; CLP; and CLP+CERA (5 µg/kg body weight, i.p. administered 24 h before CLP). At 24 hours after CLP, we measured creatinine clearance, biochemical variables, and hemodynamic parameters. In kidney tissue, we performed immunoblotting—to quantify expression of the Na-K-2Cl cotransporter (NKCC2), aquaporin 2 (AQP2), Toll-like receptor 4 (TLR4), erythropoietin receptor (EpoR), and nuclear factor kappa B (NF-κB)—and immunohistochemical staining for CD68 (macrophage infiltration). Plasma interleukin (IL)-2, IL-1β, IL-6, IL-10, interferon gamma, and tumor necrosis factor alpha were measured by multiplex detection.

**Results:**

Pretreatment with CERA preserved creatinine clearance and tubular function, as well as the expression of NKCC2 and AQP2. In addition, CERA maintained plasma lactate at normal levels, as well as preserving plasma levels of transaminases and lactate dehydrogenase. Renal expression of TLR4 and NF-κB was lower in CLP+CERA rats than in CLP rats (p<0.05 and p<0.01, respectively), as were CD68-positive cell counts (p<0.01), whereas renal EpoR expression was higher (p<0.05). Plasma levels of all measured cytokines were lower in CLP+CERA rats than in CLP rats.

**Conclusion:**

CERA protects against sepsis-induced AKI. This protective effect is, in part, attributable to suppression of the inflammatory response.

## Introduction

Severe sepsis leads to organ failure and is therefore associated with high mortality. Organ dysfunction is an independent prognostic factor for intensive care unit (ICU) mortality [1]–[3], which is highest among patients with hepatic dysfunction [4]. In patients with sepsis and concomitant acute kidney injury (AKI), ICU mortality can be as high as 70% [5]. Approximately 50% of all patients with sepsis develop AKI, which is also an independent predictor of 2-year mortality [5]–[7]. Various pathophysiological mechanisms have been proposed to explain sepsis-induced AKI [1]: vasodilation-induced glomerular hypoperfusion; dysregulated circulation within the peritubular capillary network; tubular dysfunction induced by oxidative stress; and inflammatory reactions by systemic cytokine storm or local cytokine production. In mice, a lack of anti-inflammatory proteins, such as netrin-1, has been shown to elevate renal and systemic inflammatory markers, as well as enhancing ischemia and reperfusion kidney dysfunction; in such mice, treatment with netrin-1 has been found to restore a normal phenotype during AKI [8].

Toll-like receptors (TLRs) are the major microbial pathogen receptors on innate immune cells and have been identified in various organs, including the kidney. In the kidneys of animals with sepsis, there is marked upregulation of TLR4 [9], which is known to stimulate production of the pro-inflammatory transcription factor nuclear factor kappa B (NF-κB) [10]. The activation of NF-κB plays a central role in the pathophysiology of septic shock [11], [12], inducing systemic cytokine production and thus triggering mechanisms that depress renal function in response to inflammatory diseases [13]–[16]. Mice deficient in NF-κB-dependent genes are resistant to septic shock and sepsis-related mortality [12], which indicates that inflammatory pathways play an important role in sepsis-induced organ dysfunction.

Erythropoietin is widely used as a treatment for AKI-induced anemia because it has anti-apoptotic effects on red blood cells and their precursors. It also has various extra-hematopoietic effects involving vasopressor, anti-apoptotic, anti-inflammatory and immunomodulatory activities [17]–[22]: reducing oxidative stress and lipid peroxidation; promoting renal tubular cell regeneration, vascular regeneration, and neoangiogenesis; mobilizing endothelial progenitor cells; and upregulating expression of endothelial nitric oxide synthase. In addition, erythropoietin is protective of various types of cells and tissues [23], [24], probably because erythropoietin receptors (EpoRs) are found in a variety of locations, including glomerular, mesangial, and tubular epithelial cells [24]–[26]. The intracellular domain of the EpoR contains phosphotyrosines that activate certain molecular cascades, including that involving the protein kinase B pathway, which also stimulates NF-κB activation [22], [26]. The EpoR isoforms with non-erythropoietic effects, such as tissue protection, have a lower affinity for EPO binding [22]. Therefore, the EPO dose used for this purpose should be greater than is that used in order to achieve erythropoietic effects [26].

In recent years, short-term, high-dose administration of EPO has been shown to ameliorate AKI [18], [22]–[24], [26]. Even in chronic kidney disease, treatment with recombinant human erythropoietin decreases urinary levels of protein and of biomarkers of renal injury, as well as reducing levels of markers of oxidative stress. In addition, treatment with erythropoietin lessens carotid artery intima-media thickness and reduces brachial-ankle pulse wave velocity, as well as lowering plasma levels of brain natriuretic peptide and serum levels of asymmetric dimethylarginine, both of which have been reported to be associated with cardiovascular risk factors, such as hypertension, diabetes, dyslipidemia and chronic kidney disease, and are strong predictors of cardiovascular disease and progression of chronic kidney disease [27].

Continuous erythropoietin receptor activator (CERA) is a longer-acting erythropoietin with a half-life of ≈130 h [28], compared with ≤9 h for epoetin (alfa and beta) and ≈25 h for darbepoetin alfa [29], [30]. In a nonischemic model of diabetic kidney injury in mice [31], CERA was found to protect renal function. In a model of cyclosporine A-induced renal and pancreatic islet cell injury, CERA administration correlated with increased levels of anti-inflammatory mediators—interleukin (IL)-10 and transforming growth factor beta 1—in the renal parenchyma, as well as with preserved islet cell viability [32].

The cecal ligation and puncture (CLP) model provokes polymicrobial sepsis that mimics many features of human sepsis [33]–[36]. In the present study, we used the CLP model to determine whether CERA protects against tissue damage in sepsis-induced multi-organ dysfunction, as well as to test the hypothesis that local renal TLR4 and pro-inflammatory pathways mediate sepsis-induced multi-organ dysfunction and AKI. We demonstrated that CERA is protective against sepsis-related AKI and tubular dysfunction, and that it prevents multi-organ dysfunction. Although the relationship between TLR4 and NF-κB activation remains hypothetical, we found indirect evidence that EpoR activation plays a role in sepsis-mediated inflammation.

## Results

### CERA Protects against Sepsis-Related AKI and Tubular Dysfunction

We evaluated three groups of Wistar rats, with or without CLP-induced polymicrobial sepsis and treated or not with CERA: control, consisting of untreated rats; CLP, consisting of untreated rats submitted to CLP; and CLP+CERA, consisting of rats injected with CERA (5 µg/kg body weight, i.p.) 24 h before CLP. As expected, CLP rats developed kidney dysfunction, showing, in comparison with control rats, lower urine volume and creatinine clearance, as well as higher plasma creatinine levels. In contrast, CLP+CERA rats showed higher urine volume and lower plasma creatinine, with consequently higher creatinine clearance, than did CLP rats (Table 1). Plasma levels of electrolytes (P, K, and Mg) were higher in CLP rats than in control rats and CLP+CERA rats, being comparable in the latter two groups (Table 1).

**Table 1:** Physiological parameters.

Parameter	Control	CLP	CLP+CERA
UV (ml/day)	15.5±1.8	4.6±1.5*,†	16.3±3.3
Cr (mg/dl)	0.22±0.02	0.34±0.03‖,¶	0.21±0.02
Ccr (ml • min/100 g BW)	0.73±0.05	0.32±0.10*,†	0.70±0.07
Na (mEq/L)	152±2	146±1‡	149±2
K (mEq/L)	3.8±0.1	4.8±0.3‖,¶	3.7±0.2
P (mg/dl)	7.7±0.4	9.2±0.6‡,§	7.0±0.3
Mg (mg/dl)	1.9±0.1	2.5±0.2‡	2.2±0.2
U_Osm_ (mOsm/kg)	555±28	1217±287‡,§	610±47.5
UNaV (mEq/day)	1.87±0.16	0.65±0.17‡,§	1.68±0.50
UKV (mEq/day)	0.75±0.12	0.96±0.17	0.65±0.10
UureaV (mg/day)	271.1±28	562.5±24‖,¶	210.0±29
AST (IU/L)	104±20	175±31*,†	105±7
ALT (IU/L)	37±20	70±11*,†	43±4
LDH (IU/L)	155±21	473±103†,‡	175±22
Lactate (mg/dl)	16±2	28±4*,†	12±1

CLP, cecal ligation and puncture; CERA, continuous erythropoietin receptor activator; UV, urine volume; Cr, creatinine; Ccr, creatinine clearance; BW, body weight; U_Osm_, urinary osmolality; UNaV, urinary excretion of sodium; UKV, urinary excretion of potassium; UureaV, urinary excretion of urea; AST, aspartate aminotransferase; ALT, alanine aminotransferase; LDH, lactate dehydrogenase.

*p<0.01 vs. Control; †p<0.01 vs. CLP+CERA; ‡p<0.05 vs. Control; §p<0.05 vs. CLP+CERA; ‖p<0.001 vs. Control; ¶p<0.001 vs. CLP+CERA.

As shown in Table 1, CLP rats showed significantly lower urinary excretion of sodium, as well as significantly higher urinary excretion of urea, than did control and CLP+CERA rats. There were no significant differences in terms of urinary excretion of potassium. The marked decrease in urine output in CLP rats was accompanied by increased urine osmolality, which was significantly higher than that observed for CLP+CERA rats.

**Figure 1:**
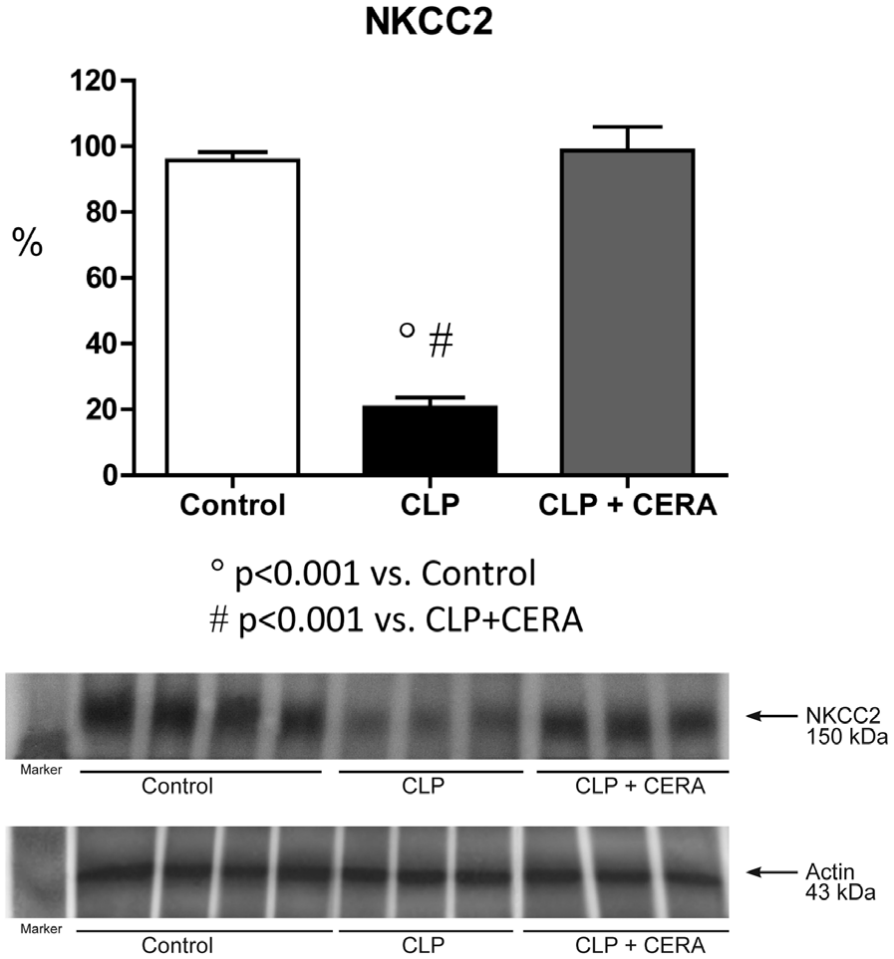
Immunoblots for renal Na-K-2Cl cotransporter, revealing a 146- to 176-kDa band (centered at 150 kDa). Semiquantitative immunoblots prepared from kidney samples. Densitometric analysis of all samples from control rats, rats submitted to cecal ligation and puncture only, and rats treated with continuous erythropoietin receptor activator prior to undergoing cecal ligation and puncture. Differences among the means were analyzed by analysis of variance followed by the Student-Newman-Keuls test. P>0.05 for control vs. CLP+CERA. CLP, cecal ligation and puncture; CERA, continuous erythropoietin receptor activator; NKCC2, renal Na-K-2Cl cotransporter.

**Figure 2:**
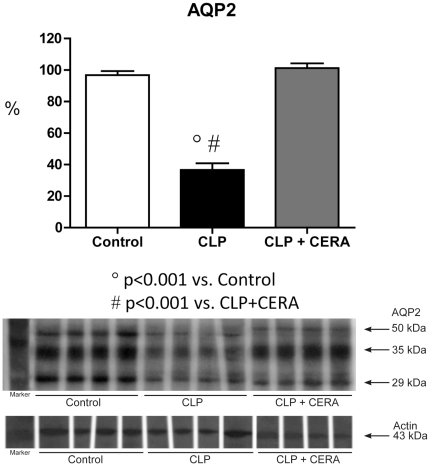
Aquaporin 2 expression was preserved in the rats pretreated with continuous erythropoietin receptor activator. Semiquantitative immunoblots prepared from kidney samples. Densitometric analysis of all samples from control rats, rats submitted to cecal ligation and puncture only, and rats treated with continuous erythropoietin receptor activator prior to undergoing cecal ligation and puncture. Differences among the means were analyzed by analysis of variance followed by the Student-Newman-Keuls test. P>0.05 for control vs. CLP+CERA. CLP, cecal ligation and puncture; CERA, continuous erythropoietin receptor activator; AQP2, aquaporin 2.

Renal expression of the Na-K-2Cl cotransporter (NKCC2) was lower in CLP rats than in CLP+CERA rats and controls (20.6%±3.1% vs. 98.7%±7.2% and 95.7%±2.5%; p<0.001; Fig. 1). Similar relationships were observed for renal aquaporin 2 (AQP2) expression (CLP vs. CLP+CERA and control: 36.7%±4.2% vs. 101.3%±3.0% and 96.8%±2.6%; p<0.001; Fig. 2).

### CERA Prevents Multi-Organ Dysfunction

In CLP+CERA rats, plasma levels of aspartate aminotransferase and alanine aminotransferase were comparable to those observed for control rats and were significantly lower than those obtained for CLP rats (Table 1). In addition, CERA administration appeared to protect the microcirculation, plasma levels of lactate and lactate dehydrogenase being significantly lower in CLP+CERA rats than in CLP rats.

### Hemodynamics

At 24 h after CLP, mean arterial pressure was similar across the groups (Table 2), nor were there any differences among the groups in terms of hematocrit values. Therefore, neither of these variables were associated with the development of or protection against AKI or multi-organ dysfunction in sepsis.

**Table 2:** Hemodynamic parameters.

Parameter	Control	CLP	CLP+CERA
Hematocrit (%)	41.7±1.3	40.6±1.8	37.7±1.8
MAP (mmHg)	115±10	111±11	115±8

CLP, cecal ligation and puncture; CERA, continuous erythropoietin receptor activator; MAP, mean arterial pressure.

### EpoR Expression

**Figure 3:**
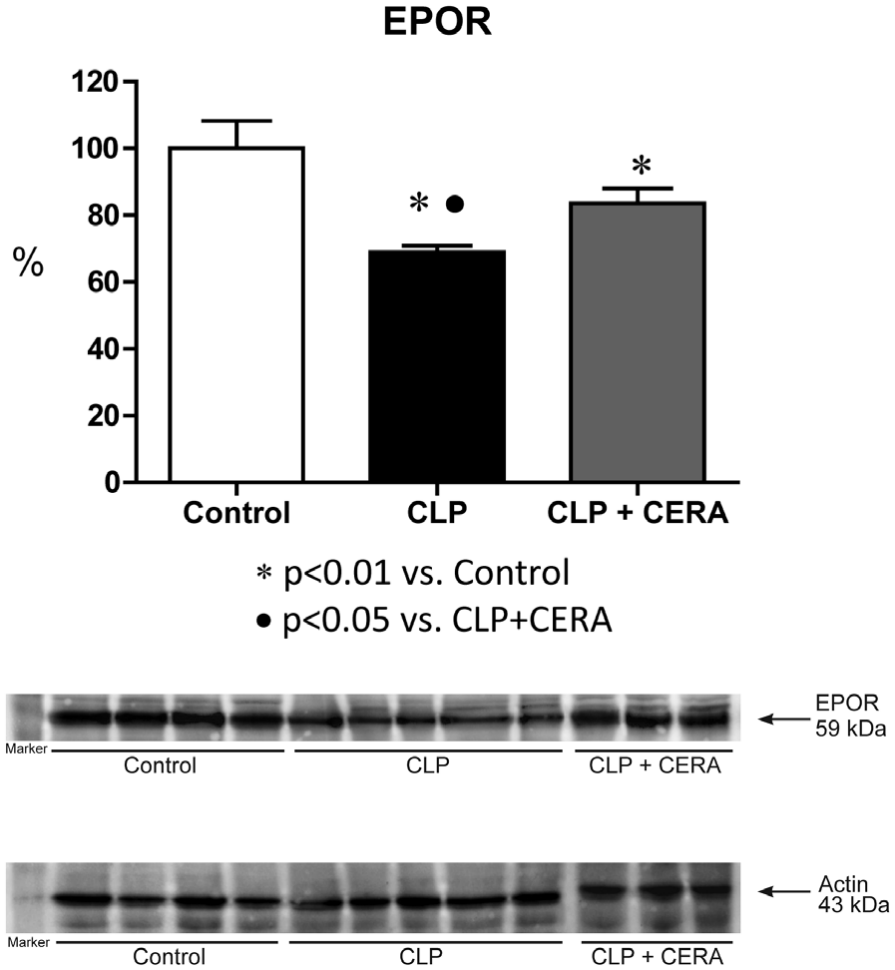
Erythropoietin receptor expression was partially protected in the rats pretreated with continuous erythropoietin receptor activator. Semiquantitative immunoblots prepared from kidney samples. Densitometric analysis of all samples from control rats, rats submitted to cecal ligation and puncture only, and rats treated with continuous erythropoietin receptor activator prior to undergoing cecal ligation and puncture. Differences among the means were analyzed by analysis of variance followed by the Student-Newman-Keuls test. p>0.05 for control vs. CLP+CERA. CLP, cecal ligation and puncture; CERA, continuous erythropoietin receptor activator; EpoR, erythropoietin receptor.

As shown in Figure 3, renal EpoR expression was markedly lower in CLP rats than in control rats (69%±2% vs. 100%±8%; p<0.01). Pretreatment with CERA partially inhibited the downregulation of EpoR (CLP+CERA: 83%±4%; p<0.05 vs. CLP).

### TLR4 and NF-κB Expression

**Figure 4:**
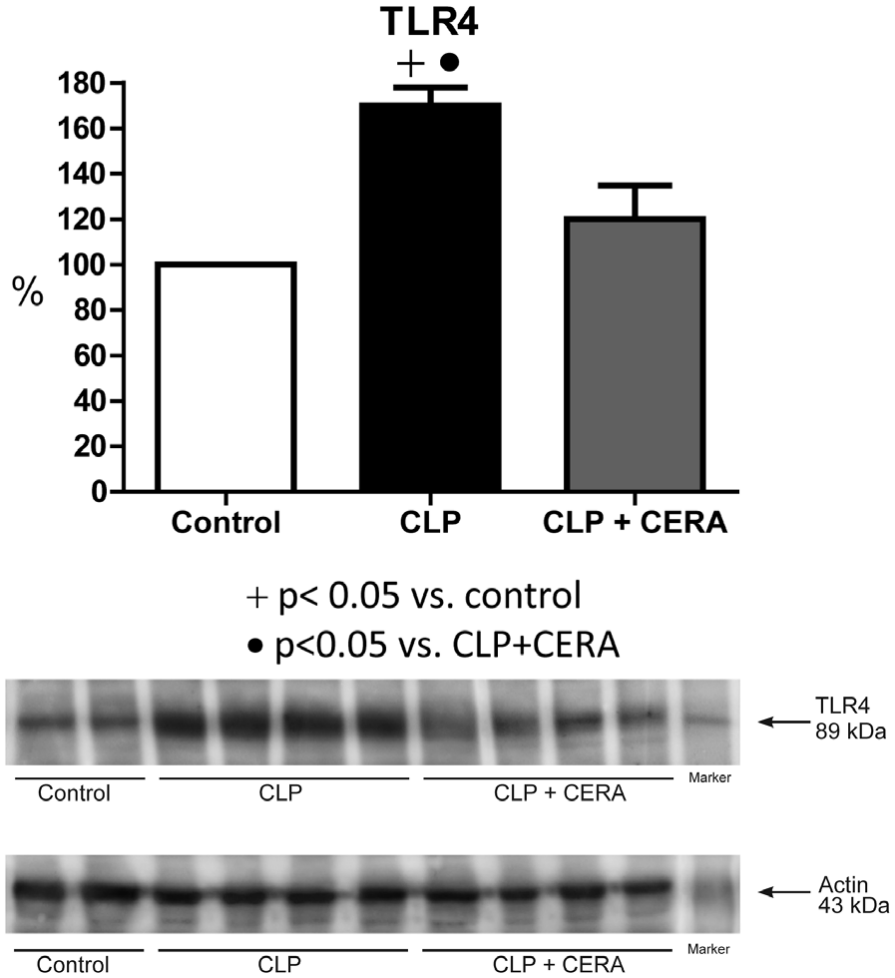
Immunoblots reacted with anti-Toll-like receptor 4 antibody (1∶100), revealing an 89-kDa band. Semiquantitative immunoblots prepared from kidney samples. Densitometric analysis of samples from control rats, rats submitted to cecal ligation and puncture only, and rats treated with continuous erythropoietin receptor activator prior to undergoing cecal ligation and puncture. Differences among the means were compared by analysis of variance followed by the Student-Newman-Keuls test. p>0.05 for control vs. CLP+CERA. CLP, cecal ligation and puncture; CERA, continuous erythropoietin receptor activator; TLR4, Toll-like receptor 4.

Figure 4 shows that renal TLR4 expression was markedly higher in the CLP group than in the control group (170%±7.9% vs. 100%±0.0%; p<0.05), whereas it was lower in the CLP+CERA group than in the CLP group (120%±14.7% vs. 170%±7.9%; p<0.05). There was no statistically significant difference between the CLP+CERA and control groups in terms of TLR4 expression.

**Figure 5:**
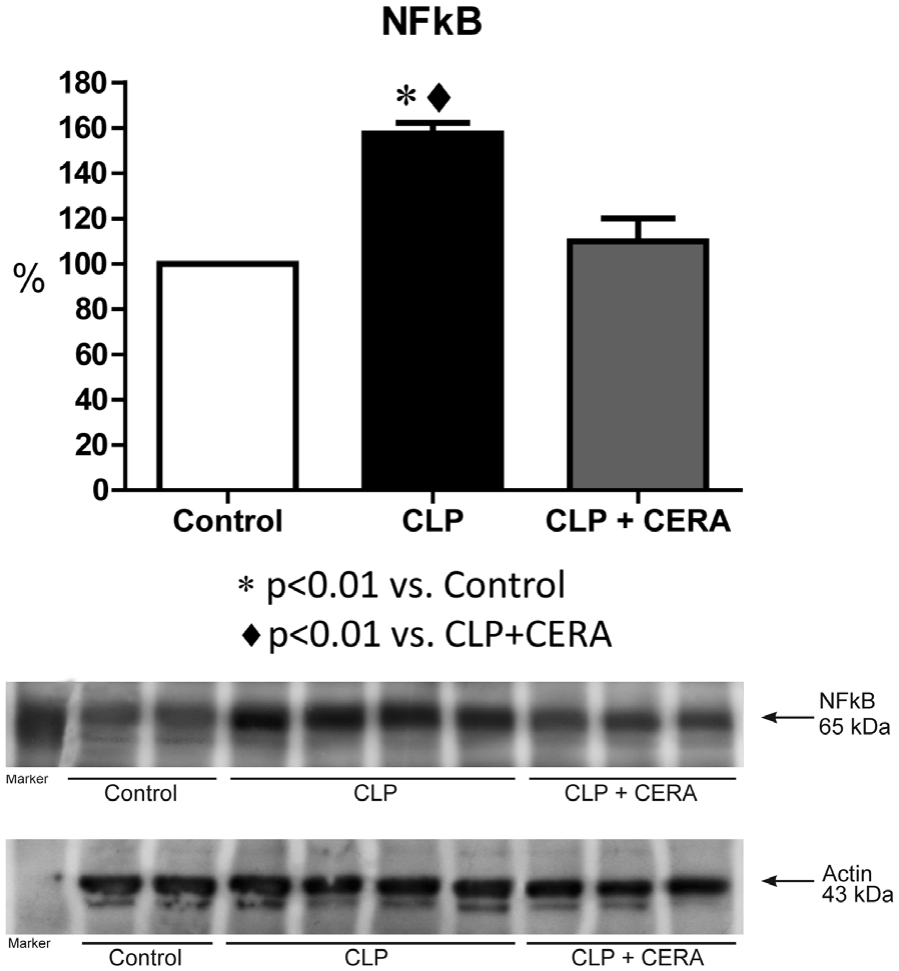
Immunoblots reacted with anti-nuclear factor kappa B antibody **(1**∶500), revealing a 65-kDa band. Semiquantitative immunoblots prepared from kidney samples. Densitometric analysis of samples from control rats, rats submitted to cecal ligation and puncture only, and rats treated with continuous erythropoietin receptor activator prior to undergoing cecal ligation and puncture. Differences among the means were compared by analysis of variance followed by the Student-Newman-Keuls test. p>0.05 for control vs. CLP+CERA. CLP, cecal ligation and puncture; CERA, continuous erythropoietin receptor activator; NF-κB, nuclear factor kappa B.

As one of the most important transcription factors in the TLR4 signaling pathway, NF-κB plays a critical role in the pathophysiology of sepsis. Figure 5 shows that, at 24 h after CLP, renal NF-κB expression was significantly higher in CLP rats than in control rats (158%±4.8% vs. 100%±0.0%; p<0.01), whereas it remained completely protected in CLP+CERA rats (110%±10.0%; p<0.01 vs. CLP).

### Inflammatory Cytokine Levels

At 24 h after CLP, plasma levels of IL-1β, IL-2, IL-6, IL-10, interferon gamma (IFN-γ), and tumor necrosis factor alpha (TNF-α) were higher in CLP rats than in control rats (Table 3). In the CLP+CERA rats, the plasma levels of these cytokines were comparable to those observed for the control animals.

**Table 3:** Effects of continuous erythropoietin receptor activator on plasma levels of pro-inflammatory cytokines at 24 h after cecal ligation and puncture.

Variable	Control	CLP	CLP+CERA
IL-1β (pg/ml)	50±12	985±399	526±193
TNF-α (pg/ml)	54±12	62±7	37±10
IFN-γ (pg/ml)	522±240	1613±631	1118±652
IL-2 (pg/ml)	36±8	74±18	29±10
IL-6 (pg/ml)	291±65	532±80*	237±44
IL-10 (pg/ml)	96±0	527±211	348±96

CLP, cecal ligation and puncture; CERA, continuous erythropoietin receptor activator; IL, interleukin; TNF-α, tumor necrosis factor alpha; IFN-γ, interferon gamma.

*p<0.05 vs. CLP+CERA.

### Macrophage Infiltration

**Figure 6:**
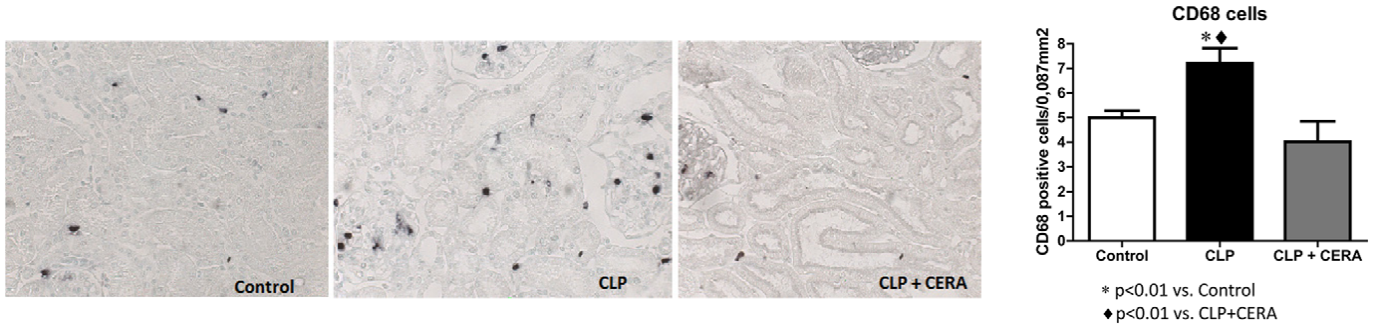
Number of CD68-positive cells/0.087 mm^2^ field in the tubulointerstitium at 24 h after CLP. **A**, photomicrographs of immunohistochemical staining in control rats, rats submitted to cecal ligation and puncture only, and rats treated with continuous erythropoietin receptor activator prior to undergoing cecal ligation and puncture (magnification, ×40). **B**, Graphic representation of CD68-positive cell counts. Data are mean±SEM. p>0.05 for control vs. CLP+CERA. CLP, cecal ligation and puncture; CERA, continuous erythropoietin receptor activator.

Infiltration of the renal interstitium by macrophages and monocytes, as quantified by the CD68-positive cell counts (Fig. 6), was significantly higher in the CLP group than in the control group (7.2±0.6 cells/mm^2^ vs. 5.0±0.3 cells/mm^2^; p<0.01). The CD68-positive cell count in the CLP+CERA group (4.0±0.8 cells/mm^2^) was significantly lower that that obtained for the CLP group (p<0.01).

## Discussion

In our experimental model of sepsis-induced multi-organ dysfunction, pretreatment with CERA had striking effects on various biologic parameters. We found that pre-CLP administration of CERA protected renal function, as well as minimizing liver injury and the damage caused by impaired microperfusion. We also demonstrated that CERA administration protected glomerular filtration against sepsis-induced impairment. In addition, tubular function (urinary excretion of sodium and urea, as well as expression of NKCC2 and AQP2) remained normal in rats pretreated with CERA. Our data indicate that CERA is renoprotective in sepsis and suggest that the mechanisms underlying cytoprotection are mediated by inflammatory responses. We also found that renal EpoR expression, which was downregulated in this model of sepsis, was preserved by pretreatment with CERA.

In rats, the CLP procedure creates a realistic model of polymicrobial sepsis [37]. The fact that patients with sepsis are also treated with fluid resuscitation and broad-spectrum antibiotics makes this model even more clinically relevant. Sepsis frequently leads to multi-organ dysfunction, which includes AKI, one of the most feared complications in patients with sepsis because it further worsens prognosis and increases the cost of care [1], [38]. It has been shown that the risk of death is 3.2 times higher in patients with AKI than in those without [7].

Previous experimental studies have also shown that erythropoietin is renoprotective in sepsis [39]. In the present study, we found that CLP decreased urinary excretion of sodium and increased urinary excretion of urea, as well as significantly increasing urinary osmolality. Semiquantitative immunoblotting showed that the expression of AQP2 and NKCC2 was decreased in untreated rats submitted to CLP. These findings confirm those of other studies, including that conducted by Grinevich et al. [13], who reported decreased AQP2 abundance in rats with endotoxemia. An increase in proximal tubule fluid reabsorption would necessarily reduce distal delivery. Whether a decrease in tubule fluid flow rate (resulting from increased proximal reabsorption) directly decreases AQP2 expression remains to be investigated. In our CLP group, despite extremely low levels of collecting duct AQP2, urine volume was low, suggesting that the flow into the collecting ducts was hindered. Therefore, we can speculate that distal delivery is indeed diminished in CLP rats, resulting in partial osmotic equilibration, despite decreased AQP2 expression. In addition, the higher urinary excretion of urea and lower urine volume resulted in greater urinary osmolality in the CLP rats.

It has been shown that the expression of AQP2 and NKCC2 in the renal outer medulla is downregulated in rats with lipopolysaccharide-induced sepsis [40]. Activated macrophages express inducible nitric oxide synthase, the inhibition of which can prevent NKCC2 downregulation in rats [41]. Therefore, the presence of activated macrophages is likely associated with the production of pro-inflammatory mediators and peroxynitrite, as well as with the regulation of AQP2 and NKCC2 [41]. In addition, it is known that injection of TNF-α, IL-1β, or IFN-γ impairs renal function and inhibits the expression of renal sodium transporters. Lipopolysaccharide-induced downregulation of sodium transporters has been shown to be unaffected in cytokine knockout mice [42]. Furthermore, Höcherl et al. [43] demonstrated that NF-κB activation plays an important role in downregulating expression of AQP2 and of vasopressin type 2 receptors in sepsis. Conversely, the authors showed that NF-κB inhibition ameliorates sepsis-induced AKI in the CLP model, which is in agreement with our finding that pre-CLP administration of CERA maintained normal expression of AQP2 and NKCC2.

It is now known that sepsis is a highly heterogeneous clinical condition. Hypotension and ischemic insult are no longer considered the main determinants of AKI. In rodents, the features of sepsis-induced AKI range from normal histology to generalized renal inflammation [42]. Renal biopsies of patients with sepsis often reveal leukocyte infiltration [1], [44]. Experimental studies have revealed that sepsis induces leukocyte infiltration into the glomerular capillaries, as well as apoptosis of glomerular endothelial cells [1], [45]. Similarly, we demonstrated that, in our CLP rats, there was considerable macrophage infiltration into the renal interstitium, and no such infiltration was observed in our control or CLP+CERA rats.

Sepsis-induced AKI seems to be an inflammatory condition in which hemodynamics play only a small role. In the present study, there were no pronounced sepsis-induced hemodynamic changes in either CLP group. Mean arterial pressure did not differ among the groups. This raises the hypothesis that hemodynamic changes play no role in CERA protection against multi-organ dysfunction.

In the present study, rats receiving pre-CLP treatment with CERA showed higher renal EpoR expression than did untreated rats submitted to CLP, suggesting that the protective effects of CERA are related to cellular EpoR activation. The pretreated rats also showed attenuated expression of TLR4.

The major physiological function of erythropoietin is the induction of erythropoiesis [39]. Anemia is quite common in critically ill patients, especially in those with AKI [46]. In such patients, erythropoiesis is impaired as a consequence of blunted erythropoietin production and the direct inhibitory effects of inflammatory cytokines [46]. One plausible hypothesis to explain this phenomenon is that cytokines diminish EpoR activation, reducing the effect of erythropoietin. However, it remains unclear whether augmented EpoR activation leads to reduced TLR4 expression or inhibition of inflammatory molecules permits higher EpoR expression.

Kalakeche et al. demonstrated that TLR4 knockout mice showed only minimal endotoxin uptake at all time points, indicating that the endotoxin pathway is TLR4-dependent [47]. Continued stimulation of TLRs can lead to immune cell apoptosis, which results in immunosuppression, a late-phase trait of sepsis [48], [49].

In animal models of septic shock, as in patients with sepsis, NF-κB activity has been found to be markedly increased in various organs [12]. In patients with sepsis, higher levels of NF-κB activity are associated with higher mortality rates and worse clinical outcomes [12]. Mice deficient in NF-κB-dependent genes are resistant to septic shock and sepsis-induced mortality [12]. Inhibition of NF-κB activation ameliorates sepsis-induced myocardial dysfunction and vascular derangement, as well as inhibiting the expression of multiple pro-inflammatory genes, reducing tissue neutrophil infiltration, and preventing microvascular endothelial leakage. Inhibition of NF-κB activation therefore prevents multi-organ dysfunction and improves survival in rodent models of septic shock [12]. Studies employing the CLP model have shown that inhibition of NF-κB activation significantly improves survival [6].

Local inflammation within the kidney is increasingly recognized as a factor contributing to inflammatory injury in remote organs [1], [50]. An AKI-induced increase in IL-6 levels triggers IL-10 production by Kupffer cells in the liver [50]–[54], resulting in kidney-liver crosstalk, a hallmark of multi-organ dysfunction. The pro-inflammatory cytokines TNF-α and IL-8 promote apoptosis via the NF-κB pathway and amplify liver injury [50], [55]–[57]. During sepsis, NF-κB is known to be activated in many organs, having potent effects on downstream signaling pathways and tissue injury [12]. Accordingly, we found that plasma levels of IL-1β, TNF-α, IFN-γ, IL-2, IL-6, and IL-10 were increased in our CLP group. In addition, there was pronounced liver injury in our CLP rats, and that injury was attenuated in the CLP+CERA rats.

Damage to the microvascular endothelium and endothelial dysfunction cause impaired perfusion in multiple organs and generate hyperlactatemia, thereby contributing to the morbidity and mortality associated with sepsis. Although hyperlactatemia is often accompanied by hemodynamic instability, it can also occur under stable hemodynamic conditions, in which case it is considered to be due to occult hypoperfusion [58]. In CLP-induced sepsis, erythropoietin can increase capillary perfusion in skeletal muscle [59]. We found that arterial lactate levels were markedly elevated in the CLP group, an alteration that was effectively prevented by pretreatment with CERA.

In the study known as the EARLYARF trial [60], erythropoietin was not found to protect against AKI in ICU patients. This discrepancy could be attributable to various factors. Patients were screened for AKI risk by determination of the urinary concentrations of two biomarkers, gamma-glutamyl transpeptidase and alkaline phosphatase. Although the gamma-glutamyl transpeptidase × alkaline phosphatase product was efficient in predicting poorer outcomes, its predictive value for AKI was poor. The conclusions regarding the development of AKI in the ICU were based on a post hoc analysis, in which all patients with AKI, regardless of the cause, were evaluated as a group. Therefore, we believe that the results of the EARLYARF trial are not definitive, and that further studies are needed in order to investigate the protective effect of erythropoietin against AKI in humans.

Our data indicate that CERA protects against multi-organ dysfunction, and that this protective effect is, in part, attributable to inhibition of the inflammatory response. We hypothesize that CERA decreases NF-κB expression via the TLR4 pathway, thereby inhibiting macrophage infiltration into the renal interstitium and decreasing systemic production of inflammatory cytokines. In addition, our findings provide indirect evidence that EpoR activation plays a role in sepsis-mediated inflammation. This could have real clinical implications for patients with sepsis, who might benefit from treatment with CERA, which has no effect on hematocrit or mean arterial pressure and could represent a feasible method of tissue protection in such patients.

## Materials and Methods

### Animals and Induction of Sepsis

The study was approved by the Research Ethics Committee of the University of São Paulo School of Medicine. All procedures were performed in accordance with the National Research Council Guide for the Care and Use of Laboratory Animals [61]. The Research Ethics Committee of Medical School of São Paulo University aproved our protocol numbered 308/10, in november 2010.

Male Wistar rats (200–250 g), obtained from the animal facility of the University of São Paulo School of Medicine, were fed a normal diet and given ad libitum access to water. The rats were divided into three groups: control (n = 11), consisting of untreated rats; CLP (n = 7), consisting of untreated rats submitted to CLP; and CLP+CERA (n = 9), consisting of rats injected with CERA (5 µg/kg body weight, i.p.; Roche Diagnostics, Penzberg, Germany) 24 h before CLP.

*The CLP procedure* involved exposing a 2-cm section of the cecum, which was ligated with a cotton suture distal to the ileocecal valve and punctured twice on its antimesenteric border with a 16-gauge needle. The cecum was then squeezed to expel a small amount of fecal material. The bowel was returned to the abdominal cavity, and the abdominal wall incision was closed. To ensure adequate fluid resuscitation, each animal received an injection of 0.15 M NaCl (25 ml/kg body weight, i.p.) immediately after the procedure. Additional fluid therapy (0.15 M NaCl, 25 ml/kg body weight, i.p.) was started at 6 h after CLP and then repeated every 12 h, as was antibiotic therapy with imipenem/cilastatin (14 mg/kg body weight, i.p.; Merck Sharp & Dohme, West Point, PA, USA).

### Metabolic Cage Studies and Hemodynamic Analysis

After CLP, all rats were moved to individual cages and maintained on a 12/12-h light/dark cycle (with ad libitum access to water only, no food provided). After 24-h urine samples had been collected, rats were anesthetized with pentobarbital sodium (50 mg/kg body weight) and placed on a temperature-regulated surgical table. A PE-60 catheter was inserted into the right carotid artery to record mean arterial pressure. Blood samples were collected by aortic puncture. Organs were then perfused with phosphate buffered saline (0.15 M NaCl and 0.01 M phosphate buffer, pH 7.4), and the kidneys were immediately removed. Some kidneys were frozen in liquid nitrogen and stored at −70°C for subsequent immunoblotting. For immunohistochemical analysis, kidneys were immersed in methacarn (60% methanol, 30% chloroform, and 10% acetic acid), after which kidney fragments were embedded in paraffin and cut into 4-µm sections.

### Analysis of Blood and Urine

Urine and blood samples were centrifuged, after which the supernatant was aliquoted and stored at −70°C for subsequently analysis. Venous plasma lactate and bicarbonate were measured with a blood gas analyzer (Radiometer Medical, Copenhagen, Denmark). Urine osmolality was determined using a vapor pressure osmometer (model 5520; Wescor, Logan, UT, USA).

We measured plasma and urinary levels of creatinine using kinetic techniques, plasma Mg^2+^ levels using automated colorimetric assay, and plasma P levels using automated enzymatic colorimetric assay. Plasma and urinary levels of other electrolytes were measured with ion-selective electrodes (NOVA Biomedical, Waltham, MA, USA). Plasma levels of liver enzymes, lactate dehydrogenase and urea were measured with automated kinetic methods.

### Antibodies and Reagents

Peptide-derived rabbit polyclonal antibody to NKCC2 was kindly supplied by Dr. M. Knepper (National Heart, Lung, and Blood Institute, National Institutes of Health, Bethesda, MD, USA). We obtained peptide-derived polyclonal antibodies to EpoR (M-20), TLR4, NF-κB, AQP2, and actin from Santa Cruz Biotechnology (Santa Cruz, CA, USA).

### Preparation of Kidney Samples

Kidney fragments were homogenized in ice-cold isolation solution (200 mM Mannitol, 80 mM Hepes, and 41 mM potassium hydroxide, pH 7.5) containing protease inhibitors (protease inhibitor cocktail; Sigma, St. Louis, MO, USA) using a Teflon pestle glass homogenizer (Schmidt and Co., Frankfurt am Main, Germany). The homogenates were centrifuged at low speed (3000 g) for 15 min at 4°C to remove nuclei and cell debris.

### Electrophoresis and Immunoblotting

Kidney samples were run on 12.5% polyacrylamide minigels (for AQP2 and EpoR), 10% polyacrylamide minigels (for NF-κB), or 8% polyacrylamide minigels (for NKCC2 and TLR4). After transfer by electroelution to nitrocellulose membranes (Hybond-P; GE Healthcare, Piscataway, NJ, USA), blots were blocked for 1 h with 5% milk and 0.1% Tween 20 in Tris-buffered saline (24.2 g/L Tris, 29.2 g/L NaCl, 3.36 g/L ethylenediaminetetraacetic acid) and then incubated overnight with the antibodies to AQP2 (1∶5000), TLR4 (1∶100), NF-κB (1∶500), NKCC2 (0.12 µg/ml), and EpoR (1∶1000). The labeling was visualized with horseradish peroxidase-conjugated secondary antibody (anti-rabbit IgG, diluted 1∶2000, and anti-goat IgG, diluted 1∶10,000, Sigma) in the enhanced chemiluminescence detection system (GE Healthcare).

### Renal Expression Analysis

The enhanced chemiluminescence films were scanned with National Institutes of Health ImageJ software. Antibody expression was quantified through densitometry, and the bands were normalized to actin expression.

### Immunohistochemistry

For CD68 immunostaining, samples were processed in 4-µm paraffin sections. After deparaffinization, endogenous peroxidase activity was blocked with 0.3% H_2_O_2_ in water for 10 min at room temperature. Sections were then subjected to incubation overnight at 4°C with CD68 antibody (1∶100). This was followed by incubation with biotinylated mouse anti-rat IgG for 30 min at room temperature. The reaction product was detected with avidin-biotin-peroxidase complex (Vector Laboratories, Burlingame, CA, USA). The color reaction was developed with 3,3-diaminobenzidine (Sigma), and the sections were counterstained with methyl green [62].

To obtain the mean numbers of infiltrating CD68-positive cells in the renal cortical tubulointerstitium, all fields (0.087 mm^2^ each) were evaluated and the mean counts per kidney were calculated.

### Cytokine Analysis

To determine plasma levels of IL-1β, IL-2, IL-6, IL-10, IFN-γ, and TNF-α, we used a Bio-Plex cytokine assay kit (Rat 9-Plex; Bio-Rad, Hercules, CA, USA). The assay was read on the Bio-Plex suspension array system, and the data were analyzed with Bio-Plex Manager software, version 4.0 [63].

### Statistical Analysis

Values are presented as means±standard error of the mean. Comparisons between groups were made by unpaired t-test. Comparisons among groups were made by analysis of variance followed by Student-Newman-Keuls multiple comparisons test. Values of p<0.05 were considered significant.

## References

[1] Chvojka J, Sýkora R, Karvunidis T, Raděj J, Kroužecký A (2010). New developments in septic acute kidney injury.. Physiol Res.

[2] Angus DC, Linde-Zwirble WT, Lidicker J, Clermont G, Carcillo J (2001). Epidemiology of severe sepsis in the United States: analysis of incidence, outcome, and associated costs of care.. Crit Care Med.

[3] Reinhart K, Meisner M, Brunkhorst FM (2006). Markers for sepsis diagnosis: what is useful?. Crit Care Clin.

[4] Umegaki T, Ikai H, Imanaka Y (2011). The impact of acute organ dysfunction on patients' mortality with severe sepsis.. J Anaesthesiol Clin Pharmacol.

[5] Bagshaw SM, Uchino S, Bellomo R, Morimatsu H, Morgera S (2007). Septic acute kidney injury in critically ill patients: clinical characteristics and outcomes.. Clin J Am Soc Nephrol.

[6] Leelahavanichkul A, Yasuda H, Doi K, Hu X, Zhou H (2008). Methyl-2-acetamidoacrylate, an ethyl pyruvate analog, decreases sepsis-induced acute kidney injury in mice.. Am J Physiol Renal Physiol.

[7] Lopes JA, Fernandes P, Jorge S, Resina C, Santos C (2010). Long-term risk of mortality after acute kidney injury in patients with sepsis: a contemporary analysis.. BMC Nephrol.

[8] Grenz A, Dalton JH, Bauerle JD, Badulak A, Ridyard D (2011). Partial netrin-1 deficiency aggravates acute kidney injury.. PLoS One.

[9] El-Achkar TM, Huang X, Plotkin Z, Sandoval RM, Rhodes GJ (2006). Sepsis induces changes in the expression and distribution of Toll-like receptor 4 in the rat kidney.. Am J Physiol Renal Physiol.

[10] Buer J, Balling R (2003). Mice, microbes and models of infection.. Nat Rev Genet.

[11] O'Sullivan AW, Wang JH, Redmond HP (2009). NF-kappaB and p38 MAPK inhibition improve survival in endotoxin shock and in a cecal ligation and puncture model of sepsis in combination with antibiotic therapy.. J Surg Res.

[12] Liu SF, Malik AB (2006). NF-kappa B activation as a pathological mechanism of septic shock and inflammation.. Am J Physiol Lung Cell Mol Physiol.

[13] Grinevich V, Knepper MA, Verbalis J, Reyes I, Aguilera G (2004). Acute endotoxemia in rats induces down-regulation of V2 vasopressin receptors and aquaporin-2 content in the kidney medulla.. Kidney Int.

[14] Escalante BA, Ferreri NR, Dunn CE, McGiff JC (1994). Cytokines affect ion transport in primary cultured thick ascending limb of Henle's loop cells.. Am J Physiol 266(6 Pt.

[15] Kohan DE (1994). Interleukin-1 regulation of collecting duct prostaglandin E2 and cyclic nucleotide accumulation.. J Lab Clin Med.

[16] Husted RF, Zhang C, Stokes JB (1998). Concerted actions of IL-1βeta inhibit Na+ absorption and stimulate anion secretion by IMCD cells.. Am J Physiol 275(6 Pt.

[17] Walden AP, Young JD, Sharples E (2010). Bench to bedside: A role for erythropoietin in sepsis.. Crit Care.

[18] Bernhardt WM, Eckardt KU (2008). Physiological basis for the use of erythropoietin in critically ill patients at risk for acute kidney injury.. Curr Opin Crit Care.

[19] Fox AC, Coopersmith CM (2009). Erythropoietin in sepsis: a new use for a familiar drug?. Crit Care Med.

[20] Santhanam AV, Smith LA, Akiyama M, Rosales AG, Bailey KR (2005). Role of endothelial NO synthase phosphorylation in cerebrovascular protective effect of recombinant erythropoietin during subarachnoid hemorrhage-induced cerebral vasospasm.. Stroke.

[21] Heeschen C, Aicher A, Lehmann R, Fichtlscherer S, Vasa M (2003). Erythropoietin is a potent physiologic stimulus for endothelial progenitor cell mobilization.. Blood.

[22] Moore E, Bellomo R (2011). Erythropoietin (EPO) in acute kidney injury. .. Ann Intensive Care.

[23] Bagnis C, Beaufils H, Jacquiaud C, Adabra Y, Jouanneau C (2001). Erythropoietin enhances recovery after cisplatin-induced acute renal failure in the rat.. Nephrol Dial Transplant.

[24] Sharples EJ, Yaqoob MM (2006). Erythropoietin in experimental acute renal failure.. Nephron Exp Nephrol.

[25] Westenfelder C, Biddle DL, Baranowski RL (1999). Human, rat, and mouse kidney cells express functional erythropoietin receptors.. Kidney Int.

[26] Johnson DW, Vesey DA, Gobe GC (2010). Erythropoietin Protects Against Acute Kidney Injury and Failure.. The Open Drug Discovery Journal.

[27] Fujiwara N, Nakamura T, Sato E, Kawagoe Y, Hikichi Y (2011). Renovascular protective effects of erythropoietin in patients with chronic kidney disease. Intern Med.

[28] Macdougall IC, Robson R, Opatrna S, Liogier X, Pannier A (2006). Pharmacokinetics and pharmacodynamics of intravenous and subcutaneous continuous erythropoietin receptor activator (C.E.R.A.) in patients with chronic kidney disease.. Clin J Am Soc Nephrol.

[29] Halstenson CE, Macres M, Katz SA, Schnieders JR, Watanabe M (1991). Comparative pharmacokinetics and pharmacodynamics of epoetin alfa and epoetin beta.. Clin Pharmacol Ther.

[30] Macdougall IC, Gray SJ, Elston O, Breen C, Jenkins B (1999). Pharmacokinetics of novel erythropoiesis stimulating protein compared with epoetin alfa in dialysis patients.. J Am Soc Nephrol.

[31] Menne J, Park JK, Shushakova N, Mengel M, Meier M (2007). The continuous erythropoietin receptor activator affects different pathways of diabetic renal injury.. J Am Soc Nephrol.

[32] Meerwein C, Korom S, Arni S, Inci I, Weder W (2011). The effect of low-dose continuous erythropoietin receptor activator in an experimental model of acute Cyclosporine A induced renal injury.. Eur J Pharmacol.

[33] Buras JA, Holzmann B, Sitkovsky M (2005). Animal models of sepsis: setting the stage.. Nat Rev Drug Discov.

[34] Wichterman KA, Baue AE, Chaudry IH (1980). Sepsis and septic shock–a review of laboratory models and a proposal.. J Surg Res.

[35] Villa P, Sartor G, Angelini M, Sironi M, Conni M (1995). Pattern of cytokines and pharmacomodulation in sepsis induced by cecal ligation and puncture compared with that induced by endotoxin.. Clin Diagn Lab Immunol.

[36] Doi K, Leelahavanichkul A, Yuen PS, Star RA (2009). Animal models of sepsis and sepsis-induced kidney injury.. J Clin Invest.

[37] Doi K, Hu X, Yuen PS, Leelahavanichkul A, Yasuda H (2008). AP214, an analogue of alpha-melanocyte-stimulating hormone, ameliorates sepsis-induced acute kidney injury and mortality.. Kidney Int.

[38] Chertow GM, Burdick E, Honour M, Bonventre JV, Bates DW (2005). Acute kidney injury, mortality, length of stay, and costs in hospitalized patients.. J Am Soc Nephrol.

[39] Fliser D, Bahlmann FH, deGroot K, Haller H (2006). Mechanisms of disease: erythropoietin–an old hormone with a new mission?. Nat Clin Pract Cardiovasc Med.

[40] Schmidt C, Höcherl K, Schweda F, Kurtz A, Bucher M (2007). Regulation of Renal Sodium Transporters during Severe Inflammation.. J Am Soc Nephrol.

[41] Olesen ET, de Seigneux S, Wang G, Lütken SC, Frøkiaer J (2009). Rapid and segmental specific dysregulation of AQP2, S256-pAQP2 and renal sodium transporters in rats with LPS-induced endotoxaemia.. Nephrol Dial Transplant..

[42] Langenberg C, Bagshaw SM, May CN, Bellomo R (2008). The histopathology of septic acute kidney injury: a systematic review.. Crit Care.

[43] Höcherl K, Schmidt C, Kurt B, Bucher M (2010). Inhibition of NF-kappaB ameliorates sepsis-induced downregulation of aquaporin-2/V2 receptor expression and acute renal failure in vivo.. Am J Physiol Renal Physiol.

[44] Lerolle N, Nochy D, Guérot E, Bruneval P, Fagon JY (2010). Histopathology of septic shock induced acute kidney injury: apoptosis and leukocytic infiltration.. Intensive Care Med.

[45] Messmer UK, Briner VA, Pfeilschifter J (1999). Tumor necrosis factor-alpha and lipopolysaccharide induce apoptotic cell death in bovine glomerular endothelial cells.. Kidney Int.

[46] du Cheyron D, Parienti JJ, Fekih-Hassen M, Daubin C, Charbonneau P (2005). Impact of anemia on outcome in critically ill patients with severe acute renal failure.. Intensive Care Med.

[47] Kalakeche R, Hato T, Rhodes G, Dunn KW, El-Achkar TM (2011). Endotoxin uptake by s1 proximal tubular segment causes oxidative stress in the downstream s2 segment.. J Am Soc Nephrol.

[48] Cohen J (2002). The immunopathogenesis of sepsis.. Nature.

[49] Hotchkiss RS, Nicholson DW (2006). Apoptosis and caspases regulate death and inflammation in sepsis.. Nat Rev Immunol.

[50] Li X, Hassoun HT, Santora R, Rabb H (2009). Organ crosstalk: the role of the kidney.. Curr Opin Crit Care.

[51] Hori O, Matsumoto M, Kuwabara K, Maeda Y, Ueda H (1996). Exposure of astrocytes to hypoxia/reoxygenation enhances expression of glucose-regulated protein 78 facilitating astrocyte release of the neuroprotective cytokine interleukin 6.. J Neurochem.

[52] Kaden J, Priesterjahn R (2000). Increasing urinary IL-6 levels announce kidney graft rejection.. Transpl Int.

[53] Deng J, Kohda Y, Chiao H, Wang Y, Hu X (2001). Interleukin-10 inhibits ischemic and cisplatin-induced acute renal injury.. Kidney Int.

[54] Daemen MA, van de Ven MW, Heineman E, Buurman WA (1999). Involvement of endogenous interleukin-10 and tumor necrosis factor-alpha in renal ischemia-reperfusion injury.. Transplantation.

[55] Locksley RM, Killeen N, Lenardo MJ (2001). The TNF and TNF receptor superfamilies: integrating mammalian biology.. Cell.

[56] Wallach D, Varfolomeev EE, Malinin NL, Goltsev YV, Kovalenko AV (1999). Tumor necrosis factor receptor and Fas signaling mechanisms.. Annu Rev Immunol.

[57] Paik YH, Schwabe RF, Bataller R, Russo MP, Jobin C (2003). Toll-like receptor 4 mediates inflammatory signaling by bacterial lipopolysaccharide in human hepatic stellate cells.. Hepatology.

[58] Jansen TC, van Bommel J, Mulder PG, Lima AP, van der Hoven B (2009). Prognostic value of blood lactate levels: does the clinical diagnosis at admission matter?. J Trauma.

[59] Kao R, Xenocostas A, Rui T, Yu P, Huang W (2007). Erythropoietin improves skeletal muscle microcirculation and tissue bioenergetics in a mouse sepsis model.. Crit Care.

[60] Endre ZH, Walker RJ, Pickering JW, Shaw GM, Frampton CM (2010). Early intervention with erythropoietin does not affect the outcome of acute kidney injury (the EARLYARF trial).. Kidney Int.

[61] Cicerone RJ, Vest CM (2011). Guide for the Care and Use of Laboratory Animals..

[62] Volpini RA, da Silva CG, Costa RS, Coimbra TM (2003). Effect of enalapril and losartan on the events that precede diabetic nephropathy in rats.. Diabetes Metab Res Rev.

[63] Correa-Costa M, Semedo P, Monteiro AP, Silva RC, Pereira RL (2010). Induction of heme oxygenase-1 can halt and even reverse renal tubule-interstitial fibrosis.. PLoS One.

